# Intelligent Building Construction Management Based on BIM Digital Twin

**DOI:** 10.1155/2021/4979249

**Published:** 2021-12-14

**Authors:** Yi Jiang

**Affiliations:** Henan Finance University, Zhengzhou 451464, China

## Abstract

In order to improve the construction effect of intelligent buildings, this paper combines the BIM digital twin technology to construct the overall structure of the building construction operation and maintenance system driven by the BIM digital twin. Moreover, this paper conducts intelligent simulation of the construction process of the building and combines the construction process of the intelligent building to apply the BIM digital twin technology to the construction management of the intelligent building. In addition, this paper uses BIM to simulate the construction process. After the construction management plan is developed, BIM can be used to simulate the construction, find the problems in the construction, and formulate a reliable construction management plan in time. Through simulation experiment research, it can be known that the intelligent building construction management model based on BIM digital twin proposed in this paper can help the deployment of intelligent building construction process in many aspects and help improve the efficiency of building construction management.

## 1. Introduction

One of the largest network interconnection tools in the world is the Internet. The Internet uses some specific technical means and facilities connecting various types of related networks well and then connects these established connections to the Internet backbone network [1]. Generally speaking, the Internet technology we are talking about is the integration and synthesis of various technologies such as network, computer technology, multimedia technology, information data exchange, and communication technology, etc., to form a systematic information technology. Internet technology can be called a universal technology without exaggeration. It is a kind of integrated information technology. Moreover, it can perform a near-real simulation restoration of various activities, things, and situations in the real world, and it can also present various concepts of people on the computer in a virtual way [2].

The construction project management system is a newly built system. Therefore, the fourth-generation development environment construction system that can quickly generate prototypes is used in development, and it can be continuously improved and improved during operation, making it an ideal project management system [3].

BIM technology, simulation analysis technology, and monitoring technology have been widely used in the construction process of buildings [4]. BIM uses all the information of the engineering project as the database of the model, which can be used for visualization of the construction plan, construction simulation, and project management during the construction phase. Simulation analysis technology can simulate the mechanical performance and deformation state of the building structure at different times during the construction process. Large-scale finite element software is usually used to realize the simulation analysis of the structure, but the analysis of complex buildings requires secondary development. Moreover, through real-time construction monitoring of the construction process, especially the important parts and key procedures, it is possible to know the stress and operating status of the structure in time during the construction process. Whether the construction monitoring technology is advanced and reasonable, plays a vital role in construction control and is also an important part of the informationization of the construction process.

## 2. Related Work

Literature [5] defined BIM as integrating building component information into the building model by means of parametric modeling. At each node in the project life cycle, project participants can transmit and exchange project information through the model. BIM is used by all parties in the industry. With the continuous development of BIM standards, the continuous research of information exchange formats, and the continuous exploration of actual projects, BIM technology has been continuously improved [6]. For the intelligent management of construction sites, literature [7] is dedicated to the development of hybrid artificial intelligence tools, which have been successfully applied in many construction industries. In particular, in the field of construction management, CAPP software is used to determine important project success factors, and the developed Evolutionary Fuzzy Hybrid Neural Network (EFHNN) is used to evaluate project success. In terms of construction site informatization management, Japan has relatively standard regulations in the implementation of construction industry informatization, and Japan has fully implemented the full life cycle informatization of construction projects. On the basis of computer-aided design (CAD), a unified IT platform is provided for interaction, and electronic drawings and diagrams are used to illustrate the construction of the project, which proves that, in the case of three-dimensional electronic drawings, the realization of visualization is the most effective [8]. Literature [9] proposed a model for designing a visual management system. According to the degree of integration of the management process, the research proposed a set of guidelines for designing and implementing a visual management system and a virtual machine practice classification method.

Literature [10] proposed an Intelligent Scheduling System (ISS); project managers can use this system to find a near-optimal scheduling plan under project goals and project constraints. The ISS system integrates most important construction factors, including schedule, cost, space, manpower, equipment, and materials, etc. and uses simulation technology to allocate resources. The system assigns different levels of priority to different activities in each simulation cycle to find a near-optimal solution, so that the final progress is closer to the optimal. There are also various tools for the development of informatization in the construction industry in Germany. One of them is called the SWLMing project, which is called the energy-saving building information management semantic Web technology.

Using BIM, the construction process can be virtualized and visualized, and the construction progress, cost, resource allocation, etc. can be simulated, and the construction plan can be continuously improved, and the rationality of the construction can be improved [11]. Literature [12] proposed and verified the method of 4D model creation and the advantages of using 4D model; the use of 4D model in construction is conducive to project schedule and control. Finally, suggestions and precautions for using BIM in the process of 4D model creation are given. Literature [13] proposes a BIM-based automatic schedule plan generation method. Managers can use the schedule information model to compile a construction schedule. The two are linked for 4D simulation to increase the practicability of the schedule and reduce the number of managers and systems in subsequent construction. Literature [14] studied the BIM-based schedule planning model, which can automatically count the activity engineering quantity and calculate the duration and then schedule the schedule according to the duration. Based on the analysis of BIM-based schedule planning and BIM functions, a schedule planning method based on BIM to build an AEC + FM integrated framework is proposed.

Literature [15] deeply studied the benefits and value of applying BIM to prepare schedule and control the schedule and finally simulated the application of BIM technology to prepare schedule. Literature [16] conceived a BIM-based schedule management system framework. On the basis of this framework, a general and detailed secondary, weekly, and daily schedule plan was formulated. This action can realize the visualization of schedule management and the basis of information integration. Collaborative optimization to achieve multiple goals enriches schedule management theory, broadens schedule management practice ideas, and has guiding significance. Literature [17] uses BIM to study the automatic generation of schedule plans, with BIM technology as the main line and foundation, and on the basis of schedule planning process analysis, it integrates the establishment of expert knowledge and experience knowledge base in the field of construction and uses rules to conduct BIM-based automatic generation of schedule. BIM can be used as a database collection of component three-dimensional geometric information and other functional information. The advantage of RFID technology is that it can track and collect component progress status information, combine the advantages of the two to complement each other, and apply it to progress management, creatively solving the real-time progress. Tracking and progress monitoring are the core issues of construction progress management [18]. Literature [19] studied the application of the Internet of Things and BIM to the construction progress of the project, through radio frequency identification, global positioning, and remote understanding, command, and scheduling of construction.

## 3. Overall Structure of Building Construction Operation and Maintenance System Driven by BIM Digital Twin

**Figure 1:**
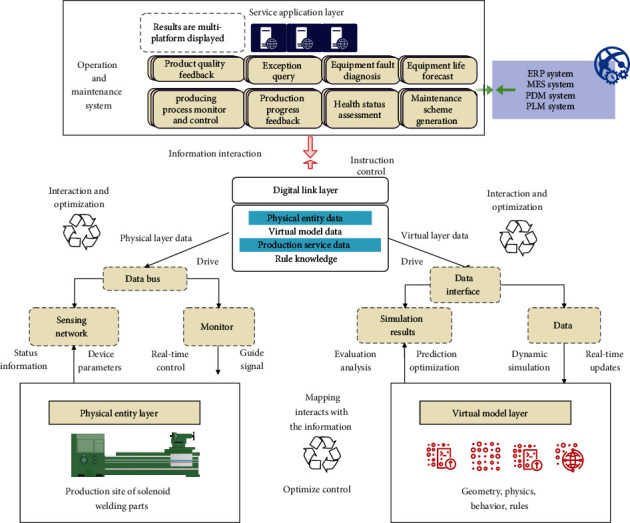
The overall structure of the construction line operation and maintenance system driven by digital twins.

Digital twin technology can be applied to a wide range of objects in the construction field, including buildings, work units, construction lines, workshops, etc. It should be noted that, in the digital twin general model architecture, when an object on the *Y*-axis (object level) is projected to the XOZ plane (system level-life cycle plane), it can be driven by the digital twin to realize the new idea of cyber-physical integration and intelligent operation of the object during its entire life cycle. Specifically, taking the building automation assembly construction line studied in this subject as an example, when it is projected along the *Y*-axis XOZ plane in the three-dimensional digital twin architecture, the overall structure of the construction line operation and maintenance system driven by the digital twin can be obtained, as shown in Figure 1.

Among them, the building construction site as a physical entity layer is the basis for realizing virtual and real interaction. During the construction process, various sensors on the construction site transmit construction status information and equipment parameters to the virtual model layer through the digital tie layer. The virtual mirroring continuously updates the data and calls the geometry, physics, behavior, and rule models to simulate the status and performance of the construction line in real time and then feeds back the simulation analysis results to the physical layer and optimizes the control of the construction site through the controller. At the same time, the data link layer transmits the construction site conditions and simulation analysis results to the service application layer. On the one hand, the service application layer integrates modules such as building quality display, construction process monitoring, abnormal situation handling, construction progress feedback, equipment fault diagnosis, health status assessment, equipment life prediction, and maintenance plan generation. It displays the operation and maintenance status of the construction line on multiple platforms. On the other hand, enterprise management information systems such as ERP, MES, PDM, and PLM will also support various functional modules in the service application layer and provide them with construction-related information through data sharing. In addition, users can issue instructions through the service application layer to optimize the control of the building construction process, which also reflects from the side that the digital twin-driven construction line operation and maintenance system have good human-computer interaction performance.

**Figure 2:**
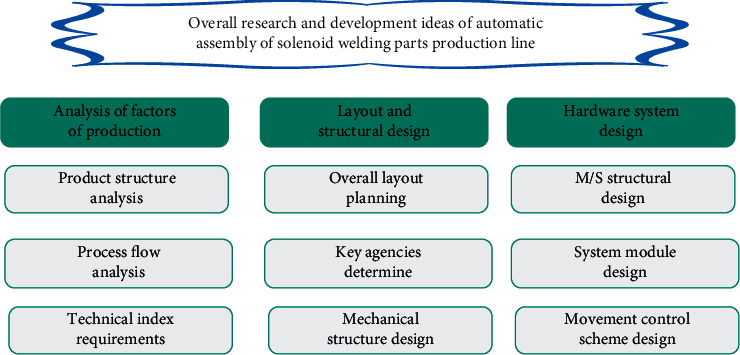
Overall research and development ideas of building automation construction line.

In building construction activities, factors such as building structure, process flow, and technical indicators are closely related to construction efficiency and quality. Therefore, the focus of research and development of the building automation construction line is to reasonably carry out construction line layout, key mechanism structure design, and control system scheme design based on the analysis of construction elements and complete the automated construction of the building through the cooperation of reasonable and effective mechanical structure and stable and reliable control system. From the three aspects of construction element analysis, layout and structural design and hardware system design, the overall research and development idea of the design and development of the building automation construction line is shown in Figure 2.

**Figure 3:**
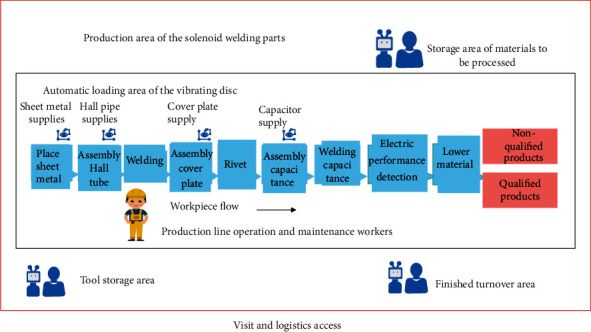
Overall layout of building automation construction line.

The overall layout of the construction line is a prerequisite for development, so the overall layout of the construction line should be combined with the current situation and needs of the enterprise. As shown in Figure 3, the construction area is divided into areas such as storage of materials to be processed, storage of tooling and tools, and construction. Among them, the building construction area includes the main construction line and the automatic feeding area, and the specific location of each station in the construction line can be determined according to the analysis of the process flow.

The virtual model of the traditional construction line mainly focuses on the visual expression, lacks the description of the characteristics, behavior, and rules of the construction line, cannot realize multidisciplinary, multiphysical, multiscale, and multiprobability simulation, and cannot meet the technical requirements of digital twins. Therefore, in the digital twin virtual model layer, researches are mainly conducted on the construction of geometric models, the standardized description of behavior models, and the evaluation of rule models.

**Figure 4:**
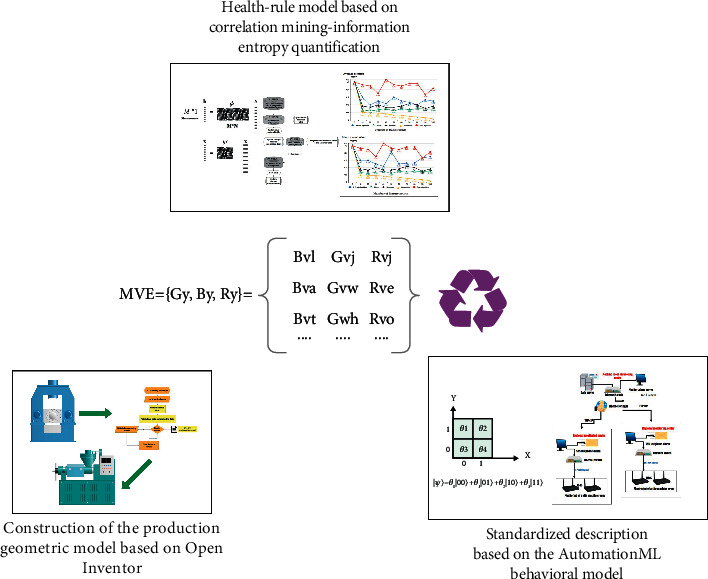
The main research content of the virtual model layer.

As shown in Figure 4, in the construction of geometric models, the expression of key attributes such as physical entity size, shape, and assembly relationship is still the focus of research. Therefore, the node information will be planned based on the analysis of the structure and movement characteristics of the key elements of the physical construction line. And then use Open Inventor to build the geometric model of the object and express it visually. In terms of behavior model description, considering that the physical construction line is a construction system that integrates multidisciplinary and multidomain knowledge, there is currently no standardized description method for behavior models, and the modeling scope of AutomationML covers multidisciplinary data information, with descriptions. The ability of the behavior model and the construction line behavior model is related to the building, resources, and technology, so the standardized description of the behavior model will be studied based on AutomationML. Since the stability and reliability of the construction line's operating state are related to the key point information, the association rules of discrete data information will be mined, and the association rules will be quantified through the information entropy method, and the construction line health rule model will be established and collected through the construction site. The temperature data analyzes and evaluates the health of the construction line to verify the accuracy of the model.

**Figure 5:**
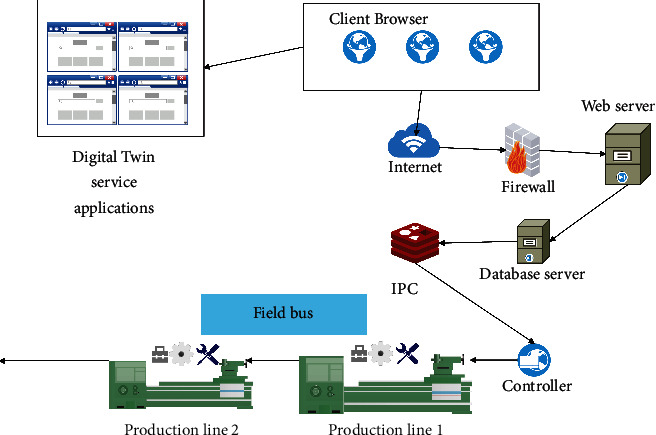
The overall scheme design of the service application layer operation and maintenance system.

At present, digital twin technology is still in its infancy, with few applications in services, and the advantages it brings are unclear. It can be seen that, through the development of the digital twin service application layer operation and maintenance system, it is very necessary to reflect the advantages of this technology in monitoring and evaluating the operation status of equipment in the construction field. The overall plan of the operation and maintenance system is shown in Figure 5. Based on the research of the physical entity layer and the virtual model layer, the key point data of the discrete construction line system is collected through the field bus to monitor the working conditions of the automated construction line at multiple construction sites. Establish a workshop Web network database server, integrate the physical entity layer construction database and the virtual model layer simulation database into the Web-core BS structure model, use the Web server to feed back the real-time construction situation and maintenance strategy of the construction site to the decision makers, and provide basic information query function. The construction information query and construction object information query functions build a unified and simple interaction method for the digital twin service application layer, physical entity layer, and virtual model layer that is independent of the user platform.

With the increase of construction line construction system equipment and the increase of uncertain factors in the construction environment, there will be complex coupling relationships within the system, which will seriously affect the reliability and stability of the construction system. Therefore, it is necessary to quantitatively evaluate the uncertain factors in the operation of the complex construction production line system. Taking into account that the digital twin rule model includes rules such as constraints, associations, and derivations, the mirroring of the virtual space can be equipped with functions such as judgment, evaluation, prediction, and optimization. Therefore, this paper defines the digital twin rule model, combines it with the health assessment of the construction line system, and uses the real-time data collected by the physical entity layer sensor as the input of the rule model. Moreover, this paper uses quantitative association rules to output health and judge, evaluate, and optimize the working status of the construction line.

When any construction system works, there will be corresponding behavior patterns, and the collection of all behavior patterns represents the construction capability of the system. Although the behavior mode of the system is the result of the interaction of different attributes, the reliability of the system operation can be obtained by analyzing it, but the complex construction system often contains more attributes with coupling relationships, which increases the difficulty of identifying the internal behavior mode. It should be noted that the real-time data collected on the construction site can effectively reflect the construction status of the resource equipment. Therefore, it is the main research direction of the digital twin rule model to obtain the health of the construction system by analyzing the real-time data stored on the construction site and quantifying the relationship between different attributes.

Since the key point monitoring data of the construction line transmitted by the digital tie layer in real time is a data sequence *x*_1_, *x*_2_, *x*_3_, ..., *x*_*n*_ that arrives continuously at a fixed speed, the sliding window model can be used to evaluate the data, that is, to analyze the collected data updated over a period of time. The data to be processed in the digital tie layer can be expressed in the form of a matrix [20]:

(1) $$\begin{array}{c} D_{DT}=\left[\begin{array}{cccc} D_{DT\;1} & D_{DT\;2} & \cdots & D_{DT\;n} \end{array}\right]=\left[\begin{array}{cccc} d_{11} & d_{12} & \cdots & d_{1n}\\ d_{21} & d_{22} & \cdots & d_{2n}\\ \vdots & \vdots & \vdots \\ d_{m1} & d_{m2} & \cdots & d_{mn} \end{array}\right]. \end{array}$$.

In the formula, Dpr represents the digital tie layer data matrix, which is composed of *n* elements representing different attributes, which can describe the temperature, force, and other attributes of the key equipment of the construction line, and the value corresponding to the attribute is represented by *d*_*mn*_. In the solenoid assembly line system, the digital tie layer data matrix mainly includes attributes such as welding temperature and hot riveting temperature.

Next, we use the digital tie layer data matrix to analyze the relationship between the data. Among the *n* attributes of the matrix (*n* > 2), two are selected arbitrarily as the *X* and *Y* axes to establish a two-dimensional scatter plot, and the extreme value in the data is used as the boundary of the two axes. At this time, a rectangular area containing all data points is obtained. According to the permutation and combination calculation formula, a data matrix with *n* attributes produces a total of *n* (*n* − 1) scatter plots. Since the data in the scatter chart represents the attributes of the object and its related operating modes, in this paper, the scatter chart is called the attribute pattern chart.

Because the continuous data stored in *D*_*DT*_ is not conducive to analysis and calculation, there is a problem of low calculation efficiency, which will affect the real-time simulation evaluation of the digital twin rule model, so these continuous attribute data need to be discretized.

**Figure 6:**
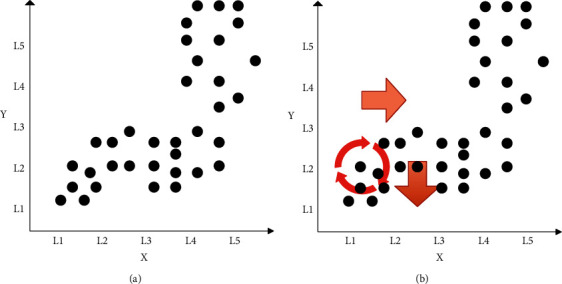
Attribute pattern diagram and association rule mining. (a) Attribute pattern diagram. (b) Association rule mining.

This paper uses the equal interval method to discretize the data and divide the data area. First, this paper determines the number of intervals to be divided. The number of intervals usually takes a value of 5–9, which represents the health assessment accuracy (EA). To facilitate analysis, the coordinate axis of the data to be analyzed is divided into five intervals, which are represented by L1, L2, L3, L4, and L5, as shown in Figure 6(a). Next, this paper conducts correlation mining on variables to study the changing trend of the system state when a certain attribute is disturbed. The figure is divided into 25 cells, and the cells containing data may represent the next operating state of the system. Therefore, the cells can be selected in turn as the reference grid, and the data can be disturbed, and the changes of other attributes can be analyzed. The mining of association rules can be expressed as [21].

(2) $$\begin{array}{c} X+\Delta X=\gg \Delta Y\left(\Delta X=\pm L\right). \end{array}$$

This formula expresses applying a unit disturbance on the *X*-axis and observing the changes on the *Y*-axis. When mining association rules, relevant indicators need to be used to determine whether the rules are valid. Here, the two concepts of confidence coefficient (Con) and support coefficient (Sup) are introduced, which, respectively, represent the degree of reliability and the degree of support. Confidence coefficient and support coefficient can be expressed as the ratio of the number of data in the reference grid to the total number of data in the current data interval, and the ratio of the number of cell data after being disturbed to the total number of data intervals occupied. In the calculation process, the minimum values of the above two coefficients are both set to 2/EA, as shown in equations (3) and (4). Taking the above attribute pattern diagram as an example, since its evaluation accuracy is 5, the minimum confidence coefficient and support coefficient are set to 40%. If the minimum confidence coefficient is met, it means that the system has enough probability to fall into this state during operation. Satisfying the minimum support coefficient indicates that, after being disturbed, the system state has enough possibility to change to another operating state.

(3) $$\begin{array}{c} \min\_\text{Con}=\frac{2}{\text{EA}}, \end{array}$$

(4) $$\begin{array}{c} \min\_\text{Sup}=\frac{2}{\text{EA}}. \end{array}$$

Taking Figure 6(a) as an example, the selected cell (2, 3) is the reference grid, which contains 8 data points, and there are 16 data points in the *X*-axis L2 interval, so the confidence coefficient is 50%. It satisfies the minimum confidence coefficient requirement; that is, during the operation, the state of the system may be the state pattern represented by the cell. When a positive disturbance is applied to the reference cell, the support coefficient of each cell on L3 is calculated, which is 6.25%, 62.5%, 31.25%, 0, 0, respectively. It can be seen that the cell (3, 2) meets the requirement of the minimum support coefficient. That is, when the system is in the state represented by cell (2, 3), if the attribute represented by the *X*-axis is disturbed in the positive direction, the system is likely to change to the state corresponding to cell (3, 2). By analogy, when negative interference is applied, the cells (1, 1) and (1, 2) meet the requirements of the support coefficient. The mining of the above association rules is shown in Figure 6(b).

After obtaining the association rules, in order to visually indicate the strength of the association rules, it needs to be quantified, and the health of the system can be obtained on this basis. Considering that entropy represents the degree of disorder of a data set and is directly related to the amount of information it contains, information entropy can be used to describe the strength of association rules. The information entropy of continuous variables can be expressed in the following form:

(5) $$\begin{array}{c} H\left(X\right)=-\int p\left(x\right)\log\;p\left(x\right)\text{d}x. \end{array}$$

However, to calculate the information entropy through this formula, the probability density function *p* (*x*) needs to be obtained first, which is more difficult in some cases, so it needs to be discretized. The information entropy after discretization is

(6) $$\begin{array}{c} H\left(X\right)=-\sum_{i=1}^{n}p\left(x_{i}\right)\log_{s}p\left(x_{i}\right). \end{array}$$

In the formula, *p*(*x*_*i*_) represents the probability when *X* takes the value xi, *i* = 1, 2,...,*n*.

In addition, mutual information represented by entropy is a useful information measure in information theory, which can measure the degree of association between two variables, and its form is shown in formula (7). Among them, the mutual information of the two variables is equal to the sum of the respective entropy minus the joint entropy. Mutual information can describe not only linear correlations between variables but also nonlinear correlations, but its values are not normalized, so mutual information needs to be normalized. This article uses generalized correlation function to quantify the relationship, such as formula (8). Among them, the value of *R*_*g*_ is between 0 and 1. The closer *R*_*g*_ is to 1, the stronger the correlation between the two attributes.

(7) $$\begin{array}{c} I\left(X,Y\right)=H\left(X\right)+H\left(Y\right)-H\left(X,Y\right), \end{array}$$

(8) $$\begin{array}{c} R_{g}=\frac{I\left(X,Y\right)}{\sqrt{H\left(X\right)H\left(Y\right)}}. \end{array}$$

When different cells are used as the reference grid, the subpatterns mined may be the same effective area, so the confidence coefficient of each reference grid needs to be considered. For a certain mode, the health degree can be expressed by the sum of the product of each submode and the confidence coefficient:

(9) $$\begin{array}{c} U\left(D_{x},D_{y}\right)=\sum_{i=1}^{EA}\sum_{j=1}^{EA}\text{con}\left(i,j\right)R_{i,j}|C_{D_{x},D_{y}}. \end{array}$$

Among them, *R*_*i*,*j*_ is the generalized correlation coefficient of the subpattern mined in the cell (*i*, *j*), and *C*_*D*_*x*_,*D*_*y*__ indicates that the calculation comes from the coordinate system composed of the Dx and Dy attributes in the digital tie layer data matrix.

For any construction system, each variable will become a benchmark attribute for analyzing the possible association relationship with other variables, and at most *n* (*n* − 1) patterns can be obtained. The collection of the above modes can reflect the operating rules of the entire system. Therefore, the system health should be a collection of all modes

(10) $$\begin{array}{c} U=\sum_{x=1}^{n}\sum_{y=1,y\neq x}^{n}U\left(D_{x},D_{y}\right)=\sum_{x=1}^{n}\sum_{y=1,y\neq x}^{n}\left(\sum_{i=1}^{EA}\sum_{j=1}^{EA}\text{con}\left(i,j\right)R_{i,j}|C_{D_{x},D_{y}}\right). \end{array}$$

## 4. Intelligent Building Construction Management Based on BIM Digital Twin

**Figure 7:**
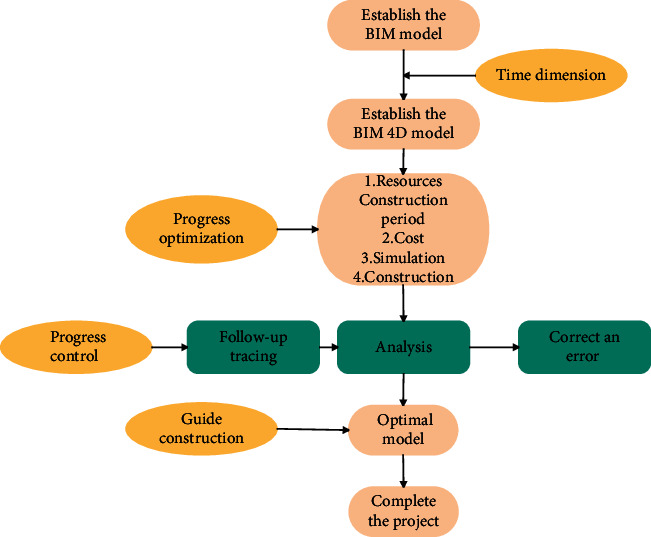
Flowchart of progress management based on BIM technology.

Compared with traditional schedule management, construction schedule management combined with BIM technology provides a communication platform that integrates multiparty information, including schedule, cost, and materials. When making schedules, it uses relevant software to simulate the project before construction to find and solve problems. During the implementation of the plan, it is possible to analyze and adjust the problems that occur efficiently and conveniently, so as to avoid missing the best time to solve the problems and affect the construction. The flowchart of the progress management based on BIM technology is shown in Figure 7.

**Figure 8:**
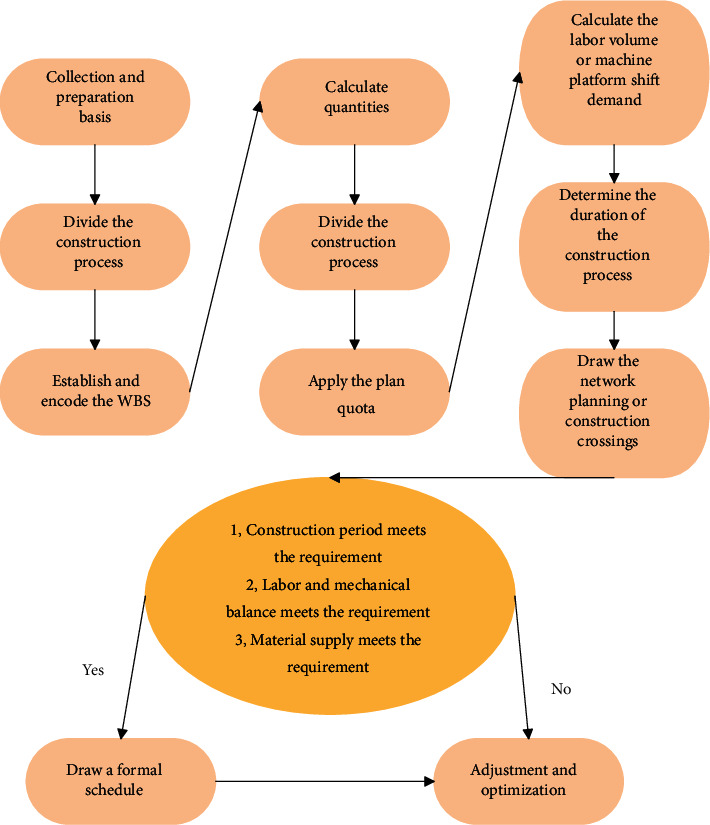
Schedule preparation process.

The schedule preparation process is shown in Figure 8.

**Figure 9:**
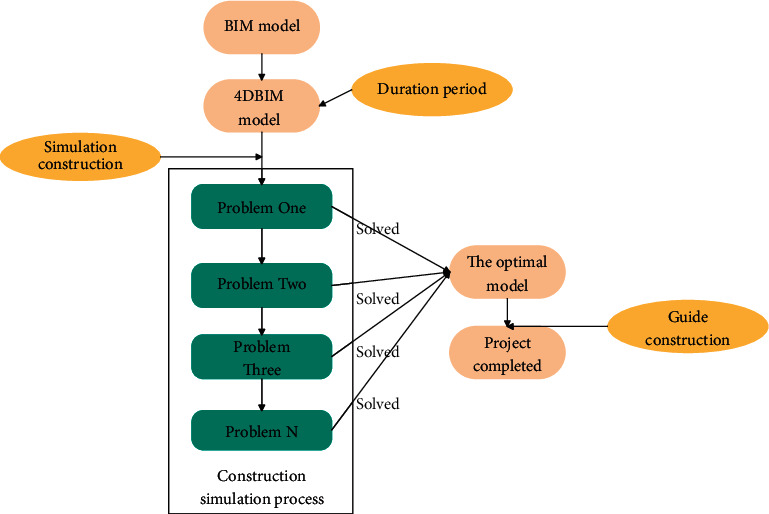
Schedule preparation and implementation procedures under BIM technology.

The schedule preparation and implementation procedure under BIM technology is shown in Figure 9 below. Under the BIM technology, through the early visual operation, the problem is first discovered, then rectified, and finally implemented.

**Figure 10:**
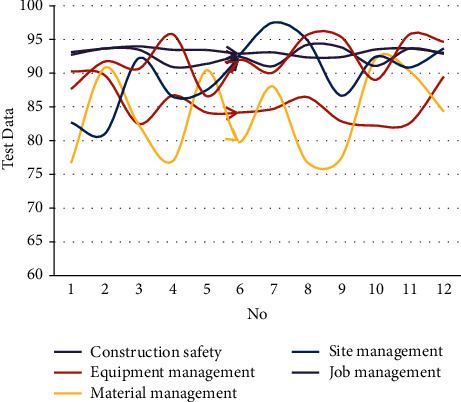
The effect of intelligent building construction management based on BIM and digital twins.

After constructing the above model, the practical effect of the intelligent building construction management model based on the BIM digital twin is studied through experiments. This article explores the effectiveness of the intelligent building construction management model based on the BIM digital twin from the aspects of construction safety, equipment management, material management, site management, operation management, and quality management. The experimental results obtained through simulation research are shown in Table 1 and Figure 10.

It can be seen from the above research that the intelligent building construction management model based on BIM digital twin proposed in this paper can help the deployment of the intelligent building construction process in many aspects and help improve the efficiency of building construction management.

## 5. Conclusion

The calculation model, material properties, construction method, construction load, temperature change, etc. of the building structure from the start to the completion stage can affect the construction quality to varying degrees. This leads to a large deviation between the actual state of the structure and the ideal state, which requires strict control of the adverse effects during the construction process. For complex structures, the mechanical properties and safety control requirements during the whole construction process are no longer what the traditional construction control technology can meet. Then, how to consider the impact of unfavorable factors on the construction status during construction and carry out real-time identification and adjustment, how to reasonably and accurately simulate the time-varying process of the structural system in each stage of construction, how to arrange the construction and schedule reasonably, and how to control the stress and strain state of the structure during construction within the allowable range are the contents and technologies that are urgently needed in the current construction field. This paper applies BIM digital twin technology to intelligent building construction management and simulates the construction process through BIM. After the construction management plan is formulated, the construction simulation can be carried out through BIM, and the problems existing in the construction can be found, and a reliable construction management plan can be formulated in time.

## Figures and Tables

**Table 1:** Simulation test results.

No	Construction safety	Equipment management	Material management	Site management	Job management	Quality control
1	93.11	90.29	76.68	82.68	92.72	87.65
2	93.65	89.66	90.82	81.02	93.64	91.72
3	93.96	82.48	82.34	92.21	93.46	90.64
4	93.45	86.70	76.97	86.53	90.92	95.75
5	93.42	84.18	90.42	87.51	91.37	86.61
6	92.90	84.16	79.76	92.49	92.44	91.91
7	93.07	84.67	88.04	97.46	91.03	90.06
8	92.34	86.46	76.92	94.99	94.17	95.62
9	92.38	82.89	77.35	86.68	93.83	95.38
10	93.49	82.23	92.06	92.41	91.08	89.00
11	93.66	82.58	90.31	90.85	93.63	95.72
12	93.00	89.50	84.30	93.66	92.86	94.63

## Data Availability

The labeled dataset used to support the findings of this study is available upon request to the author.
